# Toll-Like Receptor Signaling and Liver Fibrosis

**DOI:** 10.1155/2010/192543

**Published:** 2010-07-25

**Authors:** Tomonori Aoyama, Yong-Han Paik, Ekihiro Seki

**Affiliations:** ^1^Division of Gastroenterology, Department of Medicine, School of Medicine, University of California San Diego, 9500 Gilman Drive MC# 0702, Leichtag Biomedical Research Building Rm# 332 MM, La Jolla, CA 92093-0702, USA; ^2^Department of Internal Medicine, College of Medicine, Yonsei University, Seoul 135-720, Republic of Korea

## Abstract

Liver fibrosis occurs as a wound-healing scar response following acute and chronic liver inflammation including alcoholic liver disease, non-alcoholic steatohepatitis, hepatitis B and C, and autoimmune hepatitis. Myofibroblasts, mainly transdifferentiated from hepatic stellate cells, are pivotal cell types that produce fibrillar collagen. The activation of inflammatory cells, including Kupffer cells, is a crucial step for activating hepatic stellate cells. Toll-like receptors (TLRs) are pattern recognition receptors that sense pathogen-associated molecular patterns (PAMPs), which discriminate the products of microorganisms from the host. TLRs are expressed on Kupffer cells, endothelial cells, dendritic cells, biliary epithelial cells, hepatic stellate cells, and hepatocytes in the liver. TLR signaling induces potent innate immune responses in these cell types. The liver is constantly exposed to PAMPs, such as LPS and bacterial DNA through bacterial translocation because there is a unique anatomical link, the portal vein system between liver and intestine. Recent evidence demonstrates the role of TLRs in the activation of hepatic immune cells and stellate cells during liver fibrosis. Moreover, crosstalk between TLR4 signaling and TGF-*β* signaling in hepatic stellate cells has been reported. This paper highlights the role of TLR signaling in stellate cell activation and the progression of liver fibrosis.

## 1. Introduction

 Liver fibrosis is a wound healing scar response following acute and chronic liver diseases including chronic hepatitis B and C, autoimmune hepatitis, nonalcoholic steatohepatitis, and alcoholic liver disease [1, 2]. The pathohistological findings of liver cirrhosis, the endstage of liver fibrosis, show hepatocellular death, a lobular inflammatory cell infiltrate, excessive deposition of extracellular matrix (ECM) protein, and the appearance of regenerative nodules that may result in liver failure, portal hypertension, and hepatocellular carcinoma [1, 2]. Thus, wound healing scar response in the liver represents a harmful response rather than a beneficial response in liver regeneration. Liver fibrosis is highly associated with chronic hepatocellular injury and subsequent inflammatory response that produces inflammatory cytokines and recruits inflammatory leukocytes into the injured site. This inflammatory circumstance in the liver drives the activation of hepatic stellate cells (HSCs) through various fibrogenic mediators including TGF-*β* and PDGF [1, 2]. Activated HSCs transdifferentiate into myofiboblasts, which then produce excessive ECM proteins, including collagen type I, III, and IV. This leads to an irreversible collagen deposition, resulting in liver fibrosis [1, 2]. 

 Lipopolysaccharide (LPS, also known as endotoxin) levels in systemic and portal vein blood are increased in patients with cirrhosis [3, 4]. LPS is a Gram-negative bacterial cell wall component that binds to the pattern recognition receptor, Toll-like receptor (TLR) 4 with its coreceptors MD-2 and CD14, transmits the signals through adaptor proteins MyD88, TIRAP, TRIF, and TRAM to activate the kinases, IRAK1, IRAK4, TAK1, JNK, and IKK. These intracellular kinases lead to the activation of the transcription factors NF-*κ*B, AP-1, and interferon regulatory factors (IRFs) resulting in the induction of potent innate immune responses [5]. Kupffer cells, resident macrophages in the liver, are known to respond to LPS through TLR4 to produce various inflammatory cytokines including TNF-*α*, IL-1*β*, IL-6, IL-12, IL-18, and chemokines in granulomatous liver disease, ischemia/reperfusion liver injury, nonalcoholic steatohepatitis, and alcoholic liver disease [6–8]. HSCs, central cell types in liver fibrosis, also express high levels of TLR4 [9, 10]. A unique anatomical link, the portal vein system between the liver and intestines, may allow for the exposure of bacterial products, including LPS and bacterial DNA, to the liver [11]. However, the hepatic immune response is strictly regulated to avoid a harmful response in physiological conditions [11, 12]. In addition, sterile inflammation may provide endogenous TLR ligands for the activation of danger signals inducing fibrogenic response [7]. This paper summarizes the role of TLR signaling in HSC activation and liver fibrosis.

## 2. TLR Signaling in Hepatic Stellate Cells

HSCs are located in the space of Disse in the normal liver. Quiescent HSCs are primary cell types that store large amounts of Vitamin A-containing lipid droplets in the human body [1]. Activated HSCs are the major source of ECM protein in the fibrotic liver. Following liver injury, HSCs are activated by various fibrogenic stimuli, including TGF-*β* and PDGF, and inflammatory cytokines that are mainly produced from Kupffer cells [1, 2]. After activation, HSCs lose Vitamin A-containing lipid droplets and transdifferentiate into myofibroblasts that highly express *α*-smooth muscle actin (SMA). The excessive production and deposition of ECM proteins cause hepatic fibrosis [2]. We have demonstrated that activated human HSCs express TLR4 and its coreceptors MD-2 and CD14 [9]. LPS treatment induces the strong activation of NF-*κ*B and JNK/AP-1 pathways in activated HSCs. LPS enhances expression of the adhesion molecules ICAM-1 and VCAM-1 on the cell surface, and induces the secretion of chemokines, IL-8 and MCP-1 in activated HSCs. LPS-induced IL-8 secretion is completely blocked by inhibiting NF-*κ*B activation and partially inhibited by JNK inactivation, indicating the critical role of NF-*κ*B and JNK in TLR4 signaling of HSCs. Recently, our study demonstrated that TLR4 upregulates the expression of various types of chemokine (MCP-1, MIP-1*α*, MIP-1*β*, RANTES, KC, MIP-2, and IP-10) and TLR2, and it downregulates the expression of bone morphogenetic protein (BMP) and activin membrane bound inhibitor (Bambi), a transmembrane suppressor of TGF-*β* signaling [10, 13]. Bambi is a type I TGF-*β* receptor that lacks an intracellular kinase domain and acts as an inhibitor of BMP, activin and TGF-*β* signaling. Overexpression of Bambi inhibits, while a dominant negative form of Bambi enhances, TGF-*β* signaling in HSCs [10]. Thus, TLR4-mediated Bambi downregulation augments TGF-*β* signaling in HSCs. 

 Although the ligands for TLR3 and TLR4 stimulate HSCs to induce IFN-*β* production through adaptor TRIF in macrophages [5], HSCs could produce IFN-*β* in response to the ligand for TLR3, but not TLR4, suggesting unique TLR3/TLR4-TRIF signaling pathways in HSCs, which might be distinct from those in macrophages [14]. 

 HSCs express TLR2, a receptor for Gram-positive bacterial cell wall components, such as peptidoglycan and lipoteichoic acid [5, 15]. HSCs barely respond to TLR2 ligands. Pretreatment of TNF-*α* or IL-1*β* significantly upregulates TLR2 expression in HSCs. This primes HSCs to increase NF-*κ*B activation and IL-8 production in response to TLR2 ligands [16]. LPS also upregulates TLR2 expression in HSCs [10], suggesting that the initiation by inflammatory mediators such as TNF-*α*, IL-1*β*, and LPS might be required for fulfilling TLR2 signaling in HSCs.

 TLR9 that recognizes bacteria-derived, unmethylated CpG-containing DNA, is expressed in HSCs [17]. Watanabe et al. has demonstrated that host-derived denatured DNA from apoptotic hepatocytes induces a differentiation of HSC via TLR9 [17]. Apoptotic hepatocyte DNA induces fibrogenic responses with the elevation of mRNA levels of TGF-*β* and collagen type I in HSCs. In addition, apoptotic hepatocyte-derived DNA inhibits PDGF-induced HSC chemotaxis through TLR9 and MyD88 [17].

## 3. TLR4 Signaling in Liver Fibrosis

The activation of both HSCs and Kupffer cells that express TLR4 is associated with the progression of liver fibrosis. TLR4-mutant mice have less liver inflammation and fibrosis than TLR4-wild-type mice following bile duct ligation (BDL) and chronic treatment of carbon tetrachloride (CCl_4_), or thioacetamide [10]. Mice deficient in CD14 and LPS-binding protein also show decreased cholestasis-induced liver fibrosis [18]. These results suggest a strong contribution of LPS-TLR4 interaction in the development of liver fibrosis. Indeed, systemic plasma LPS levels are significantly elevated in these three mouse models of experimental liver fibrosis [10, 19, 20], suggesting that intestinal microflora-derived LPS translocates into the liver through the portal vein by increased intestinal permeability following liver injury. We have tested the contribution of intestinal microflora in liver fibrosis. Mice were orally treated with a cocktail of nonabsorbable broad-spectrum antibiotics (ampicillin, neomycin, metronidazole, and vancomycin) for 4 weeks prior to induction of liver fibrosis [10, 21]. This antibiotic cocktail successfully reduced plasma LPS levels after BDL, leading to a significant attenuation of liver inflammation and fibrosis [10]. Thus, intestinal microflora-derived and translocated LPS participate in TLR4-mediated liver fibrosis, most likely due to increased intestinal permeability induced by intestinal dysbiosis, such as bacterial overgrowth, and disintegrity in the tight junction of intestinal epithelium. TLR4 is also activated by endogenous ligands, such as HMGB1, hyaluronan, and heat shock protein 60 [15, 22, 23]. Currently, we do not have strong evidence that endogenous TLR4 ligands are involved in liver fibrosis. Further investigation is needed. 

 Kupffer cells are well-known targets for TLR4 ligand LPS and produce various types of inflammatory and fibrogenic cytokines, which may activate HSCs [6, 7]. Quiescent and activated HSCs also express TLR4 [9, 10]. The specific roles of TLR4 in Kupffer cells and HSCs during liver fibrosis were unknown. To investigate these roles, we generated TLR4-chimeric mice by using bone marrow (BM) transplantation (BMT) [10]. Kupffer cells are known as radio-resistant cells [24, 25]. Thus, a standard type of BMT with whole body irradiation insufficiently replaces Kupffer cells with donor BM-derived cells. To resolve this problem, we have established a new style of BM-chimera using a combination of whole body irradiation and BMT with specific deletion of Kupffer cells by liposomal clodronate injection [10]. These TLR4-chimeric mice have a successful replacement of endogenous Kupffer cells with donor BM-originated Kupffer cells, which contain TLR4-mutant BM-derived hematopoietic cells, including Kupffer cells, and TLR4-wild recipient-originated endogenous liver cells, including hepatocytes and HSCs. Few hepatocytes induce NF-*κ*B nucleartranslocation in response to LPS in Kupffer cell-depleted mice, confirming that hepatocytes barely respond to the ligand for TLR4 compared with nonparenchymal liver cells [10, 26]. Recent exclusive studies have confirmed that HSCs are not BM derived [27, 28]. As mentioned above, Kupffer cells and HSCs are direct targets of LPS in vitro and in vivo [6, 9, 10]. The specific roles of TLR4 in Kupffer cells and HSCs were discriminated by this TLR4-chimera system. In this study, the mice with TLR4-mutant endogenous liver cells exhibited a significant reduction of liver fibrosis, and the mice with TLR4-wild endogenous liver cells had a sufficient degree of fibrosis in the liver after BDL [10]. These findings indicate that the recipient-originated endogenous liver cells, but not donor-derived BM cells including Kupffer cells, are crucial cell types that respond to TLR4 ligands in liver fibrosis.

## 4. Crosstalk between TLR4 Signaling and TGF-*β* Signaling in Stellate Cell Activation

There are at least two roles of TLR4 signaling in HSCs. First, TLR4-stimulated HSCs produce various chemokines and express adhesion molecules (ICAM-1, VCAM-1, and E-selectin) to recruit Kupffer cells and/or circulating macrophages by the site of HSCs. Indeed, conditioned medium produced from LPS-treated HSCs increased Kupffer cell migration and adhesion [10]. Second, the activation of TLR4 signaling enhances TGF-*β* signaling in HSCs [10]. HSCs isolated from collagen promoter-driven GFP transgenic (Coll-GFP) mice increase GFP intensity when collagen promoter activity is increased [29]. TGF-*β* treatment alone slightly increased collagen promoter activity in quiescent HSCs whereas LPS pretreatment further increased TGF-*β*-induced collagen promoter activity in HSCs. These findings suggested that TLR4 signaling enhances TGF-*β* signaling in HSCs [10]. We then tested the role of Kupffer cells in HSC activation by co-cultured Coll-GFP HSCs with Kupffer cells. Coll-GFP HSCs cocultured with Kupffer cells increased GFP expression, which was further augmented by LPS pretreatment, suggesting that TLR4 signaling enhances Kupffer cell-mediated HSC activation [10]. Coll-GFP HSCs co-cultured with TLR4-mutant Kupffer cells express a similar level of GFP expression to the HSCs co-cultured with wild-type Kupffer cells after LPS stimulation. Thus, TLR4 on Kupffer cells has a minor role for TLR4-mediated HSC activation, but Kupffer cells are required for HSC activation as the important source of TGF-*β* because HSC activation was completely abolished by treatment with a TGF-*β* inhibitor in co-culture of Kupffer cells and HSCs. 

 Comprehensive microarray analysis demonstrated that Bambi, a transmembrane TGF-*β* receptor inhibitor, was downregulated in HSCs after LPS stimulation, whereas other TGF-*β* signaling associated genes (TGF-*β* receptor, Smad family, SNoN, Ski, and Sara) were unchanged [10, 13]. Quiescent HSCs, but not Kupffer cells and hepatocytes, express high levels of Bambi in the liver. Importantly, Bambi expression is suppressed in in vivo activated HSCs isolated from mice after BDL or chronic CCl_4_ treatment whereas HSCs isolated from bile duct ligated-TLR4-mutant mice have unchanged levels of Bambi expression [10]. We suggest that high levels of Bambi expression restrict TGF-*β* signaling in HSCs of normal livers. Upon TLR4 stimulation Bambi expression is quickly decreased. Then, TGF-*β* signaling becomes free from the restriction by Bambi to promote fibrogenic response. Interestingly, HSCs activated in culture do not downregulate Bambi expression. Thus, Bambi downregulation is an important feature of HSC activation in vivo. One study clearly demonstrated that human Bambi inhibits TGF-*β*-induced Smad3 phosphorylation, and silencing endogenous Bambi enhances TGF-*β* reporter activity [30]. The study further revealed that Bambi interacts with Smad7, interfering with the complex composed of type I and type II TGF-*β* receptors, and Smad3, resulting in inhibiting TGF-*β* signaling. In addition, Harada et al. demonstrated that Bambi expression in biliary epithelial cells is downregulated during epithelial-mesenchymal transition (EMT), suggesting an additional role of Bambi as a marker for EMT [31].

**Figure 1:**
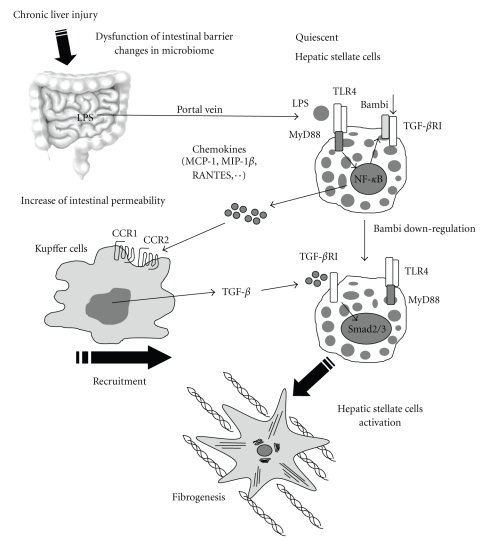
TLR4 signaling enhances TGF-*β* signaling in hepatic stellate cells. Upon liver injury, intestinal permeability is increased due to the intestinal dysbiosis and tight junction disintegrity, which allows microflora-derived LPS into the portal vein. This LPS stimulates TLR4 on hepatic stellate cells (HSCs). Quiescent HSCs express high levels of Bambi which restricts TGF-*β* signaling. TLR4 stimulation leads to the production of various chemokines (MCP-1, MIP-1*β*, and RANTES) in HSCs, recruiting Kupffer cells through their CCR1 and CCR2. Recruited Kupffer cells produce TGF-*β* which binds to TGF-*β* receptor type I in HSCs. Simultaneously, TLR4 signaling downregulates Bambi expression through MyD88 and NF-*κ*B in HSCs. HSCs become free from restricted TGF-*β* signaling by the downregulation of Bambi, which eventually induces HSC activation.

 TLR4 signaling activates NF-*κ*B and JNK/AP-1 pathways through MyD88 and TRIF [15]. TLR4-mediated downregulation of Bambi expression requires the activation of NF-*κ*B and partially JNK through MyD88, but not TRIF, in HSCs. Indeed, Bambi expression was downregulated in WT and TRIF^−/−^ HSCs, but not in MyD88^−/−^, NF-*κ*B, or JNK inactivated HSCs after LPS stimulation (E.S. unpublished observation) [10]. Similarly, MyD88^−/−^, but not TRIF^−/−^, mice demonstrated reduced fibrogenic gene expression at the early phase after BDL, whereas both MyD88^−/−^ and TRIF^−/−^ mice had reduced liver fibrosis at the late phase of liver fibrosis [10]. These findings suggest that MyD88 is crucial for the Bambi-regulated liver fibrosis, whereas TRIF regulates fibrogenic responses independently of Bambi, at least at the chronic stage. Taken together, TLR4 signaling is crucial in the activation of HSCs during liver fibrosis. Intestinal microflora is a major source of LPS as a ligand for TLR4 in liver fibrosis. TLR4 signaling in HSCs enhances the recruitment of inflammatory cells and downregulates Bambi for fibrogenic response Figure 1.

## 5. TLR4 Polymorphism, HSCs, and Liver Fibrosis

A recent genecentric functional genome scan in patients with chronic hepatitis C virus has identified seven single nucleotide polymorphisms (SNPs) that may predict the risk of developing liver cirrhosis [32, 33]. Among these seven SNPs, a TLR4 T399I SNP is the second most predictive in protecting the progression of liver cirrhosis. TLR4 D299G is another TLR4 SNP. These two SNPs are associated with a blunted response to LPS [34]. These findings confirmed the relevance of TLR4 in a large group of patients with liver fibrosis. Based on this study, Guo et al. examined the response of HSCs with TLR4 D299G and T3991 SNPs to LPS. TLR4 D299G and/or T3991 SNPs were reconstituted into a human stellate cell line, LX-2 cells, and immortalized TLR4^−/−^ mouse HSCs (TLR4^−/−^mHSC) [35]. LX-2 cells or TLR4^−/−^mHSCs expressing either the one or both SNPs displayed a marked reduction of NF-*κ*B activity and cytokine production (MCP-1 and IL-6) and unchanged Bambi expression [35]. The TLR4 SNPs inhibited HSC growth and enhanced spontaneous HSC apoptosis [35]. These findings demonstrated the mechanistic function of the two TLR4 SNPs in HSC activation and the risk of fibrosis progression.

## 6. TLR3 in Liver Fibrosis

TLR3 recognizes double stranded RNA, such as polyinosinic-polycytidylic acid (poly I:C), to induce a potent innate immune response that includes the production of interferon type I and type II. TLR3 ligand poly I:C treatment attenuates liver fibrosis induced by the treatment of 3,5-diethoxycarbonyl-1,4-dehydrocollidine (DDC) diet and CCl_4_ [36]. This fibrosis reduction by poly I:C is NK cell dependent and IFN-*γ* dependent. NK cells stimulated with poly I:C induce the cytotoxicity to activated HSCs, but not quiescent HSCs. Poly I:C directly, and indirectly through IFN-*γ*, enhances TRAIL expression in NK cells, and this increases the cytotoxicity of NK cells against activated HSCs, resulting in the suppression of liver fibrosis. The research group further demonstrated that poly I:C-dependent NK cell-dependent suppression of liver fibrosis is inhibited in ethanol-treated animals [37]. These findings suggest that the reduction of TLR3-mediated NK cell-dependent HSC killing is one of the mechanisms underlying the enhancement of liver fibrosis in alcoholic liver disease.

## 7. TLR9 in Liver Fibrosis

TLR9 recognizes bacteria-derived unmethylated CpG rich DNA [5]. A recent report from our group clearly suggests that liver fibrosis progression is associated with intestinal microflora-derived products and their translocation [10]. Previous studies have shown that patients and animals with cirrhosis have increased bacterial DNA levels in their plasma and ascites [38, 39]. On the other hand, Watanabe et al. demonstrated that denatured host origin DNA from dying hepatocytes stimulates HSCs through TLR9 in liver fibrosis. TLR9^−/−^ mice showed a reduction of liver fibrosis after BDL and chronic CCl_4_ treatment [17, 40]. These findings suggest that both bacterial DNA and host denatured DNA derived from dying cells participate in the progression of liver fibrosis as a ligand for TLR9. 

 Recently, Connolly et al. have demonstrated the contribution of TLR9 and CD11c-expressing cells to liver fibrosis [41]. CD11c is expressed on dendritic cells (DCs) and some monocytes and macrophages [42]. They used the model of liver fibrosis induced by thioacetamide plus leptin treatment as a similar pathophysiology to CCl_4_ models. Depletion of CD11c-expressing cells using CD11c-DTR mice significantly reduced liver fibrosis, suggesting the importance of CD11c-expressing non-parenchymal liver cells in the progression of liver fibrosis [41]. CD11c-expressing cells from fibrotic liver increased the capacity to produce TNF-*α*, IL-6, and various chemokines, which were further increased by TLR9 ligand stimulation in CD11c-expressing cells of fibrotic livers, but not of normal livers. This increased sensitivity of fibrotic liver CD11c-positive cells to TLR ligands was induced only by the ligand for TLR9, not TLR3, TLR4, or TLR5. CpG-DNA-primed fibrotic liver CD11c-positive cells increased NK cell cytotoxicity and cytokine production, including IFN-*γ* in a TNF-*α* dependent manner [41]. CpG-DNA-primed fibrotic liver CD11c-positive cells induced HSC proliferation and production of inflammatory mediators, such as IL-1*α*, IL-6, and MCP-1. This study demonstrated the important features of TLR9 and DCs in liver fibrosis using CD11c-depleted mice. However, the studies for DCs in liver fibrosis are still incomplete. Further studies are needed to clarify the role of DCs in the progression of liver fibrosis.

## 8. TAK1 in Liver Fibrosis

**Figure 2:**
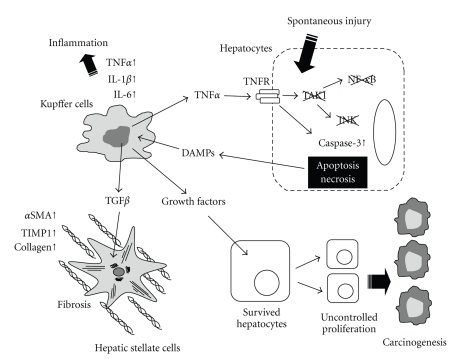
Ablation of TAK1 in hepatocytes induces spontaneous liver injury, inflammation, fibrosis, and cancer. Spontaneous hepatocyte death occurs in hepatocyte specific TAK1-deficient mice followed by the release of damage-associated molecular patterns (DAMPs) which stimulate Kupffer cells to produce TNF-*α*. This TNF-*α* further induces cell death in TAK1-deficient hepatocytes lacking activation of NF-*κ*B and JNK. TNF-*α*, IL-1*β*, and IL-6 released from Kupffer cells cause liver inflammation. Kupffer cell-derived TGF-*β* stimulates hepatic stellate cells resulting in fibrogenesis. The persistent hepatocyte death and uncontrolled compensatory proliferation in the livers of hepatocyte specific TAK1-deficient mice induce the reactivation of onco-fetal liver genes that are associated with the initiation of hepatic carcinogenesis.

TAK1 is a MAP3K which is activated by the signaling of TLRs, IL-1 receptor, TNF receptor, and TGF-*β* receptor [43, 44]. TAK1 is an upstream kinase of both IKK/NF-*κ*B and JNK/AP-1 pathways [15]. The NF-*κ*B pathway regulates the expression of antiapoptotic genes, such as Bcl-2, Bcl-xL, A20, iNOS, c-FLIP, IAPs, and TRAF family molecules, to block death receptor-mediated or mitochondria-mediated hepatocytes death [45]. NF-*κ*B also prevents prolonged JNK activation. Prolonged JNK activation induces phosphorylation of the E3 ligase Itch and subsequent ubiquitination and degradation of caspase-8 inhibitor c-FLIP, which accelerates hepatocyte apoptosis [46]. Thus, NF-*κ*B protects hepatocytes from apoptosis whereas JNK promotes apoptosis. Therefore, we could not predict whether TAK1 tends to induce or protect from hepatocyte apoptosis. As expected, neither NF-*κ*B nor JNK activation following TNF-*α* stimulation occurred in TAK1^−/−^ hepatocytes [47]. Surprisingly, TAK1^−/−^ hepatocytes increased the sensitivity to TNF-*α*-induced cell death. More surprisingly, spontaneous hepatocyte death occurred in hepatocyte specific TAK1^−/−^ mice [47]. These mice display spontaneous liver injury, inflammation, and fibrosis at the age of one month and develop hepatocellular carcinoma at the age of nine months [47, 48]. These results suggest that spontaneous persistent hepatocyte death occurs in hepatocyte specific TAK1^−/−^ mice, and these dying hepatocytes release alarmins which stimulate both Kupffer cells and HSCs, resulting in liver inflammation and fibrosis Figure 2. These findings are evidence that liver fibrosis and carcinogenesis are associated with persistent hepatocyte injury and inflammation without any carcinogens. Hepatocyte specific TAK1^−/−^ mice will be great animal models for addressing the role of the interplay between fibrosis and hepatocellular carcinoma.

## 9. Conclusion

 While patients with mild to moderate liver fibrosis may not show clinical symptoms, liver cirrhosis is a major cause of morbidity and mortality. Recent advanced studies demonstrated the strong evidence of the role of TLR signaling in liver fibrosis. A number of issues concerning the role of TLRs for HSC activation and liver fibrosis still need to be addressed. First, although we have shown the evidence that intestinal microflora promotes liver fibrogenesis due to bacterial translocation, we still need to address the mechanism of bacterial translocation in liver fibrosis, which includes changes in microbiome composition and disintegrity of intestinal tight junction. Second, the studies for the endogenous TLR ligands which promote HSC activation have not been completed. Very recently, mitochondrial DNA has been reported as an endogenous TLR9 ligand [49], which might be an endogenous ligand to activate HSCs. Third, we need to address the mechanism by which TLR signaling regulates Bambi expression and by which Bambi regulates HSC activation. The research on TLRs and HSCs in liver fibrosis has just been started. The future basic and translational studies will uncover additional clinical relevance of TLRs and their related signaling in liver fibrosis. Clinically, hepatocellular carcinoma is strongly associated with liver fibrosis and cirrhosis. However, it is still unknown whether severe liver fibrosis promotes the initiation and/or progression of hepatocellular carcinoma. We wish to open this mysterious door in future studies using hepatocyte specific TAK1^−/−^ mice.

##  Conflicts of Interest

There is no conflict of interest to disclose for all authors.

## References

[1] Bataller R, Brenner DA (2005). Liver fibrosis. *Journal of Clinical Investigation*.

[2] Friedman SL (2008). Mechanisms of hepatic fibrogenesis. *Gastroenterology*.

[3] Lin R-S, Lee F-Y, Lee S-D (1995). Endotoxemia in patients with chronic liver diseases: relationship to severity of liver diseases, presence of esophageal varices, and hyperdynamic circulation. *Journal of Hepatology*.

[4] Chan CC, Hwang SJ, Lee FY (1997). Prognostic value of plasma endotoxin levels in patients with cirrhosis. *Scandinavian Journal of Gastroenterology*.

[5] Takeda K, Akira S (2005). Toll-like receptors in innate immunity. *International Immunology*.

[6] Seki E, Tsutsui H, Nakano H (2001). Lipopolysaccharide-induced IL-18 secretion from murine Kupffer cells independently of myeloid differentiation factor 88 that is critically involved in induction of production of IL-12 and IL-1*β*. *Journal of Immunology*.

[7] Seki E, Brenner DA (2008). Toll-like receptors and adaptor molecules in liver disease: update. *Hepatology*.

[8] Szabo G, Dolganiuc A, Mandrekar P (2006). Pattern recognition receptors: a contemporary view on liver diseases. *Hepatology*.

[9] Paik Y-H, Schwabe RF, Bataller R, Russo MP, Jobin C, Brenner DA (2003). Toll-like receptor 4 mediates inflammatory signaling by bacterial lipopolysaccharide in human hepatic stellate cells. *Hepatology*.

[10] Seki E, De Minicis S, Österreicher CH (2007). TLR4 enhances TGF-*β* signaling and hepatic fibrosis. *Nature Medicine*.

[11] Crispe IN (2009). The liver as a lymphoid organ. *Annual Review of Immunology*.

[12] Crispe IN (2003). Hepatic T cells and liver tolerance. *Nature Reviews Immunology*.

[13] Onichtchouk D, Chen Y-G, Dosch R (1999). Silencing of TGF-*β* signalling by the pseudoreceptor BAMBI. *Nature*.

[14] Wang B, Trippler M, Pei R (2009). Toll-like receptor activated human and murine hepatic stellate cells are potent regulators of hepatitis C virus replication. *Journal of Hepatology*.

[15] Akira S, Uematsu S, Takeuchi O (2006). Pathogen recognition and innate immunity. *Cell*.

[16] Paik Y-H, Lee KS, Lee HJ (2006). Hepatic stellate cells primed with cytokines upregulate inflammation in response to peptidoglycan or lipoteichoic acid. *Laboratory Investigation*.

[17] Watanabe A, Hashmi A, Gomes DA (2007). Apoptotic hepatocyte DNA inhibits hepatic stellate cell chemotaxis via toll-like receptor 9. *Hepatology*.

[18] Isayama F, Hines IN, Kremer M (2006). LPS signaling enhances hepatic fibrogenesis caused by experimental cholestasis in mice. *American Journal of Physiology—Gastrointestinal and Liver Physiology*.

[19] Nolan JP, Leibowitz AI (1978). Endotoxin and the liver. III. Modification of acute carbon tetrachloride injury by polymyxin b—an antiendotoxin. *Gastroenterology*.

[20] Grinko I, Geerts A, Wisse E (1995). Experimental biliary fibrosis correlates with increased numbers of fat-storing and Kupffer cells, and portal endotoxemia. *Journal of Hepatology*.

[21] Rakoff-Nahoum S, Paglino J, Eslami-Varzaneh F, Edberg S, Medzhitov R (2004). Recognition of commensal microflora by toll-like receptors is required for intestinal homeostasis. *Cell*.

[22] Tsung A, Sahai R, Tanaka H (2005). The nuclear factor HMGB1 mediates hepatic injury after murine liver ischemia-reperfusion. *Journal of Experimental Medicine*.

[23] Schwabe RF, Seki E, Brenner DA (2006). Toll-like receptor signaling in the liver. *Gastroenterology*.

[24] Klein I, Cornejo JC, Polakos NK (2007). Kupffer cell heterogeneity: functional properties of bone marrow-derived and sessile hepatic macrophages. *Blood*.

[25] Kennedy DW, Abkowitz JL (1997). Kinetics of central nervous system microglial and macrophage engraftment: analysis using a transgenic bone marrow transplantation model. *Blood*.

[26] Isogawa M, Robek MD, Furuichi Y, Chisari FV (2005). Toll-like receptor signaling inhibits hepatitis B virus replication in vivo. *Journal of Virology*.

[27] Kisseleva T, Uchinami H, Feirt N (2006). Bone marrow-derived fibrocytes participate in pathogenesis of liver fibrosis. *Journal of Hepatology*.

[28] Higashiyama R, Moro T, Nakao S (2009). Negligible contribution of bone marrow-derived cells to collagen production during hepatic fibrogenesis in mice. *Gastroenterology*.

[29] Magness ST, Bataller R, Yang L, Brenner DA (2004). A dual reporter gene transgenic mouse demonstrates heterogeneity in hepatic fibrogenic cell populations. *Hepatology*.

[30] Yan X, Lin Z, Chen F (2009). Human BAMBI cooperates with Smad7 to inhibit transforming growth factor-*β* signaling. *Journal of Biological Chemistry*.

[31] Harada K, Sato Y, Ikeda H (2009). Epithelial-mesenchymal transition induced by biliary innate immunity contributes to the sclerosing cholangiopathy of biliary atresia. *Journal of Pathology*.

[32] Huang H, Shiffman ML, Friedman S (2007). A 7 gene signature identifies the risk of developing cirrhosis in patients with chronic hepatitis C. *Hepatology*.

[33] Li Y, Chang M, Abar O (2009). Multiple variants in toll-like receptor 4 gene modulate risk of liver fibrosis in Caucasians with chronic hepatitis C infection. *Journal of Hepatology*.

[34] Arbour NC, Lorenz E, Schutte BC (2000). TLR4 mutations are associated with endotoxin hyporesponsiveness in humans. *Nature Genetics*.

[35] Guo J, Loke J, Zheng F (2009). Functional linkage of cirrhosis-predictive single nucleotide polymorphisms of toll-like receptor 4 to hepatic stellate cell responses. *Hepatology*.

[36] Radaeva S, Sun R, Jaruga B, Nguyen VT, Tian Z, Gao B (2006). Natural killer cells ameliorate liver fibrosis by killing activated stellate cells in NKG2D-dependent and tumor necrosis factor-related apoptosis-inducing ligand-dependent manners. *Gastroenterology*.

[37] Jeong W, Park O, Gao B (2008). Abrogation of the antifibrotic effects of natural killer cells/interferon-*γ* contributes to alcohol acceleration of liver fibrosis. *Gastroenterology*.

[38] Francés R, Benlloch S, Zapater P (2004). A sequential study of serum bacterial DNA in patients with advanced cirrhosis and ascites. *Hepatology*.

[39] Guarner C, González-Navajas JM, Sánchez E (2006). The detection of bacterial DNA in blood of rats with CCl 4-induced cirrhosis with ascites represents episodes of bacterial translocation. *Hepatology*.

[40] Gäbele E, Mühlbauer M, Dorn C (2008). Role of TLR9 in hepatic stellate cells and experimental liver fibrosis. *Biochemical and Biophysical Research Communications*.

[41] Connolly MK, Bedrosian AS, Mallen-St. Clair J (2009). In liver fibrosis, dendritic cells govern hepatic inflammation in mice via TNF-*α*. *Journal of Clinical Investigation*.

[42] Aloman C, Tacke F (2010). Dendritic cells in liver fibrosis: conductor of the inflammatory orchestra?. *Hepatology*.

[43] Shim J-H, Xiao C, Paschal AE (2005). TAK1, but not TAB1 or TAB2, plays an essential role in multiple signaling pathways in vivo. *Genes and Development*.

[44] Sato S, Sanjo H, Takeda K (2005). Essential function for the kinase TAK1 in innate and adaptive immune responses. *Nature Immunology*.

[45] Schwabe RF, Brenner DA (2006). Mechanisms of liver injury. I. TNF-*α*-induced liver injury: role of IKK, JNK, and ROS pathways. *American Journal of Physiology-Gastrointestinal and Liver Physiology*.

[46] Chang L, Kamata H, Solinas G (2006). The E3 ubiquitin ligase itch couples JNK activation to TNF*α*-induced cell death by inducing c-FLIPL turnover. *Cell*.

[47] Inokuchi S, Aoyama T, Miura K (2010). Disruption of TAK1 in hepatocytes causes hepatic injury, inflammation, fibrosis, and carcinogenesis. *Proceedings of the National Academy of Sciences of the United States of America*.

[48] Bettermann K, Vucur M, Haybaeck J (2010). TAK1 suppresses a NEMO-dependent but NF-kappaB-independent pathway to liver cancer. *Cancer Cell*.

[49] Zhang Q, Raoof M, Chen Y (2010). Circulating mitochondrial DAMPs cause inflammatory responses to injury. *Nature*.

